# Supplementary material for: Association between oral microbiome and seven types of cancers in East Asian population: a two-sample Mendelian randomization analysis
Source: Front Mol Biosci. 2023 Nov 21;10:1327893. doi: 10.3389/fmolb.2023.1327893 (PMC10702768; doi:10.3389/fmolb.2023.1327893)
Supplement: Supplementary file 1 [file DataSheet1.ZIP › Supplementary Materials/MR plots for tongue/tongue═╝/Colorectal cancer/pheno.417_to_colorectal cancer_leave_one_out.pdf]

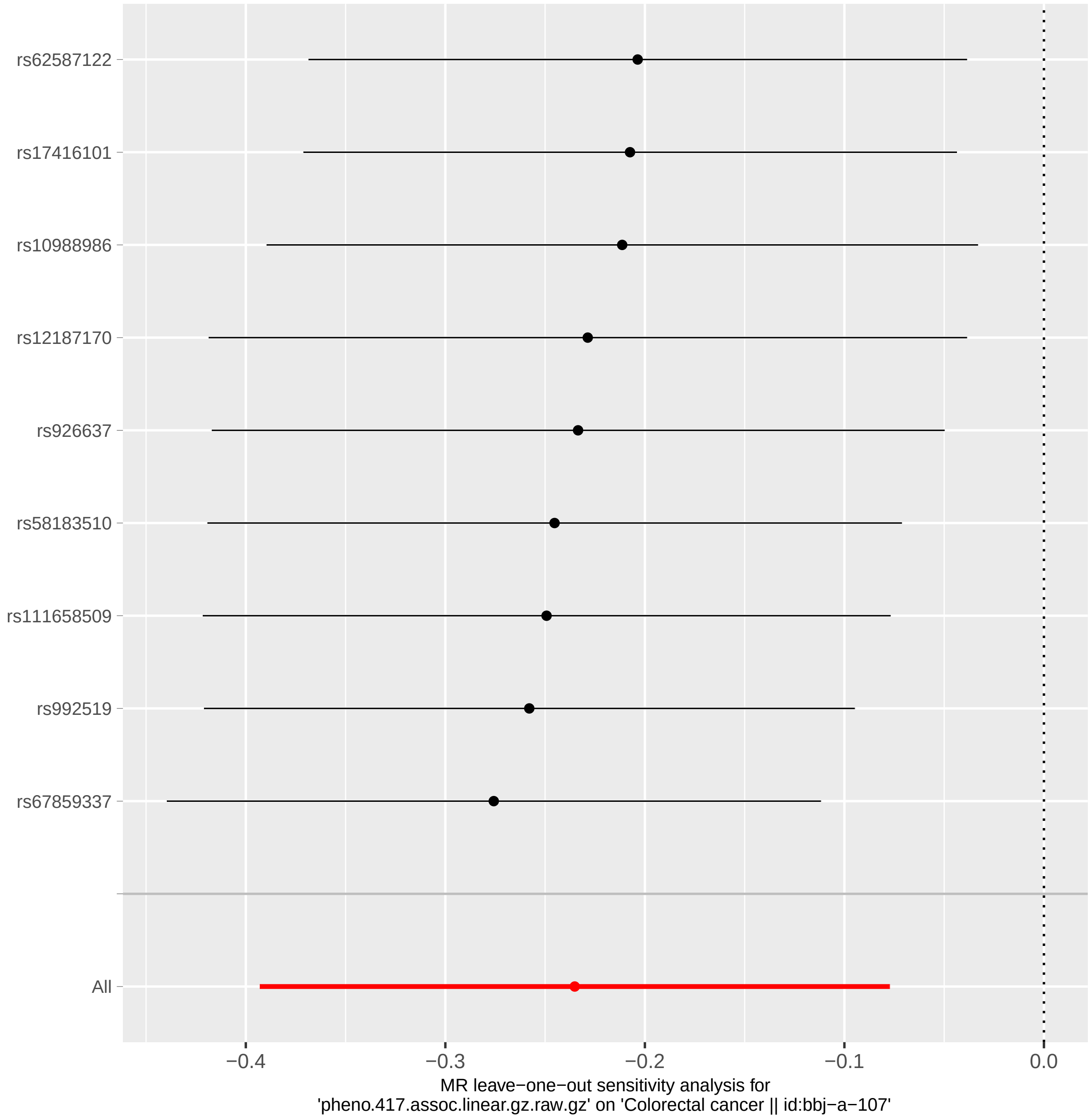
rs62587122
rs17416101
rs10988986
rs12187170
rs926637
rs58183510
rs111658509
rs992519
rs67859337
All
−0.4
−0.3
−0.2
−0.1
0.0
MR leave−one−out sensitivity analysis for
'pheno.417.assoc.linear.gz.raw.gz' on 'Colorectal cancer || id:bbj−a−107'
